# Misdiagnosis analysis and multidisciplinary collaborative treatment of primary cutaneous diffuse large B-cell lymphoma: case report

**DOI:** 10.3389/fmed.2026.1852379

**Published:** 2026-06-26

**Authors:** Hongjuan Ji, Qiao Zhang, Gaowei Zhang, Hongli Ji, Lei Zhao

**Affiliations:** 1 Department of Ultrasound Medicine, The 988th Hospital of Joint Logistic Support Force of the Chinese People's Liberation Army, Zhengzhou, Henan, China; 2 Department of Hematology and Oncology, The 988th Hospital of Joint Logistic Support Force of the Chinese People's Liberation Army, Zhengzhou, Henan, China; 3 Department of Orthopedics, The 988th Hospital of Joint Logistic Support Force of the Chinese People's Liberation Army, Zhengzhou, Henan, China

**Keywords:** diffuse large B-cell lymphoma, lymphoma, primary cutaneous malignant lymphoma, rituximab, skin

## Abstract

Primary cutaneous diffuse large B-cell lymphoma (PC-DLBCL) is a rare subtype of non-Hodgkin lymphoma with nonspecific cutaneous manifestations, leading to frequent misdiagnosis. This case describes an elderly patient with severe ulcerative skin lesions who was misdiagnosed repeatedly and required multidisciplinary orthopedic–hematologic management, thus providing valuable evidence for clinical practice. An 85-year-old male presented with progressive erythematous papules, ulcers, and subcutaneous nodules on the right forearm, chest, back, and left upper arm. Pathology and immunohistochemistry confirmed primary cutaneous diffuse large B-cell lymphoma. He underwent orthopedic debridement, autologous skin grafting, and 4 cycles of R-miniCHOP immunochemotherapy. Skin lesions resolved completely without recurrence, and 7-month follow-up showed favorable recovery. The key take-away is that PC-DLBCL is often misdiagnosed; early pathological biopsy and multidisciplinary collaboration between orthopedics and hematology-oncology are essential for accurate diagnosis and effective treatment to improve prognosis in elderly patients.

## 1 Introduction

Lymphoma currently ranks the 10th in the incidence of malignant tumors in China according to statistical data (1), and B-cell non-Hodgkin’s lymphoma (NHL) accounts for the majority of clinical cases (2). Primary cutaneous diffuse large B-cell lymphoma (PC-DLBCL) is a rare subtype of non-Hodgkin lymphoma with nonspecific cutaneous manifestations, leading to substantial misdiagnosis in clinical practice (3). It predominantly affects elderly patients and progresses rapidly, often resulting in severe skin ulceration and secondary tissue damage when treatment is mismanaged or delayed. This case is rare because it involves an 85-year-old male who was repeatedly misdiagnosed with skin allergy, skin cancer, and pemphigus at local clinics, leading to severe ulceration, infection, and unbearable pain that required orthopedic intervention before oncological therapy. Unlike typical reports that focus solely on dermatological or hematological management, this case highlights the critical value of multidisciplinary collaboration between orthopedics and hematology-oncology. By combining surgical debridement, autologous skin grafting, and R-miniCHOP immunochemotherapy, satisfactory clinical outcomes were achieved. This report adds to the literature by emphasizing early pathological biopsy and multidisciplinary care as key strategies to reduce misdiagnosis, avoid iatrogenic injury, and improve prognosis for elderly patients with advanced ulcerative PC-DLBCL.

## 2 Case presentation

### 2.1 Clinical data

An 85-year-old male patient presented with erythematous papules on the ulnar side of the right forearm in July 2023. The papules protruded from the skin surface with a scattered blister-like appearance, then coalesced into a crater-like shape accompanied by local redness, swelling and persistent pain. The patient was initially diagnosed with “skin allergy” in a local clinic and treated with topical antiallergic drugs and plasters, with poor clinical efficacy. In November 2023, the patient received topical plasters and local bloodletting therapy in a local hospital, which led to gradual ulceration and exudation of the local skin, with significantly aggravated pain at night. The patient received repeated diagnosis and treatment in local community clinics and county-level general hospitals from July 2023 to Jan 2024. No diagnostic skin biopsy was arranged in all previous outside medical visits, all diagnoses were empirical clinical diagnosis without pathological confirmation, repeated topical medication and bloodletting therapy accelerated skin ulceration. He was admitted to the Department of Orthopedics of our hospital for standardized treatment on January 25, 2024.

*Skin physical examination*: a 7 cm × 5 cm wound was found on the ulnar side of the right forearm, with a central ulcer accompanied by redness, swelling and exudation; the wound had a firm texture with scattered black crusts on the surface, dark red infiltrative edges extending to the surrounding normal skin, and scattered tension blisters around the edges with slightly elevated local skin temperature. Multiple nodules of varying sizes protruding from the skin surface were observed on the anterior chest, back and left upper arm, with central ulceration and surrounding scattered blisters arranged in circular or elliptical shapes.

*Admission laboratory tests showed abnormal indicators as follows*: creatinine 139.0 μmol/L (↑); Serum β2-microglobulin 3.3 mg/L (↑); D-dimer 0.53 mg/L (↑); Glycosylated hemoglobin 11.61% (↑); Fasting blood glucose 18.98 mmol/L (↑); Rheumatoid factor 52.6 IU/mL (↑); Anti-streptolysin O (ASO) 25.0 IU/mL.

### 2.2 Pathological diagnosis

**Figure 1:**
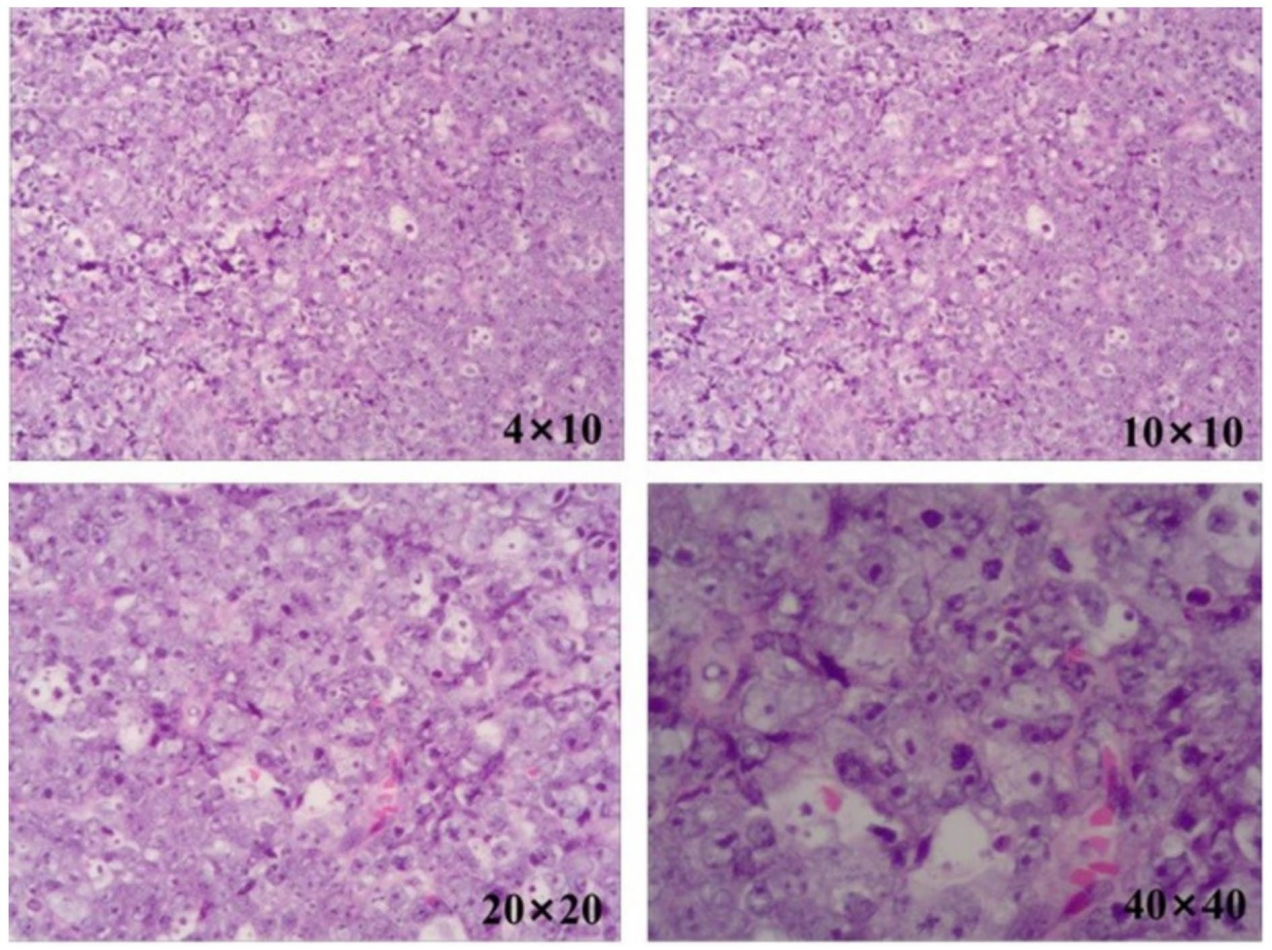
HE staining of the ulcerated skin tissue (4 × 10, 10 × 10, 20 × 20, 40 × 40 magnification). The pathological section shows the morphological characteristics of tumor cells in the ulcerated skin tissue.

On January 29, 2024, the patient underwent debridement and lesion resection of the right upper extremity under brachial plexus block anesthesia. Postoperative pathological examination of the resected tissue showed: Residual tumor cells were found at the basal margin of the ulcerated skin tissue; no definite residual lesions were found at the four skin margins of the peritumoral skin tissue; suspicious residual tumor cells were found in the deep fascial layer lesion tissue, pending further confirmation by routine paraffin sections (Figure 1, HE staining).

**Figure 2:**
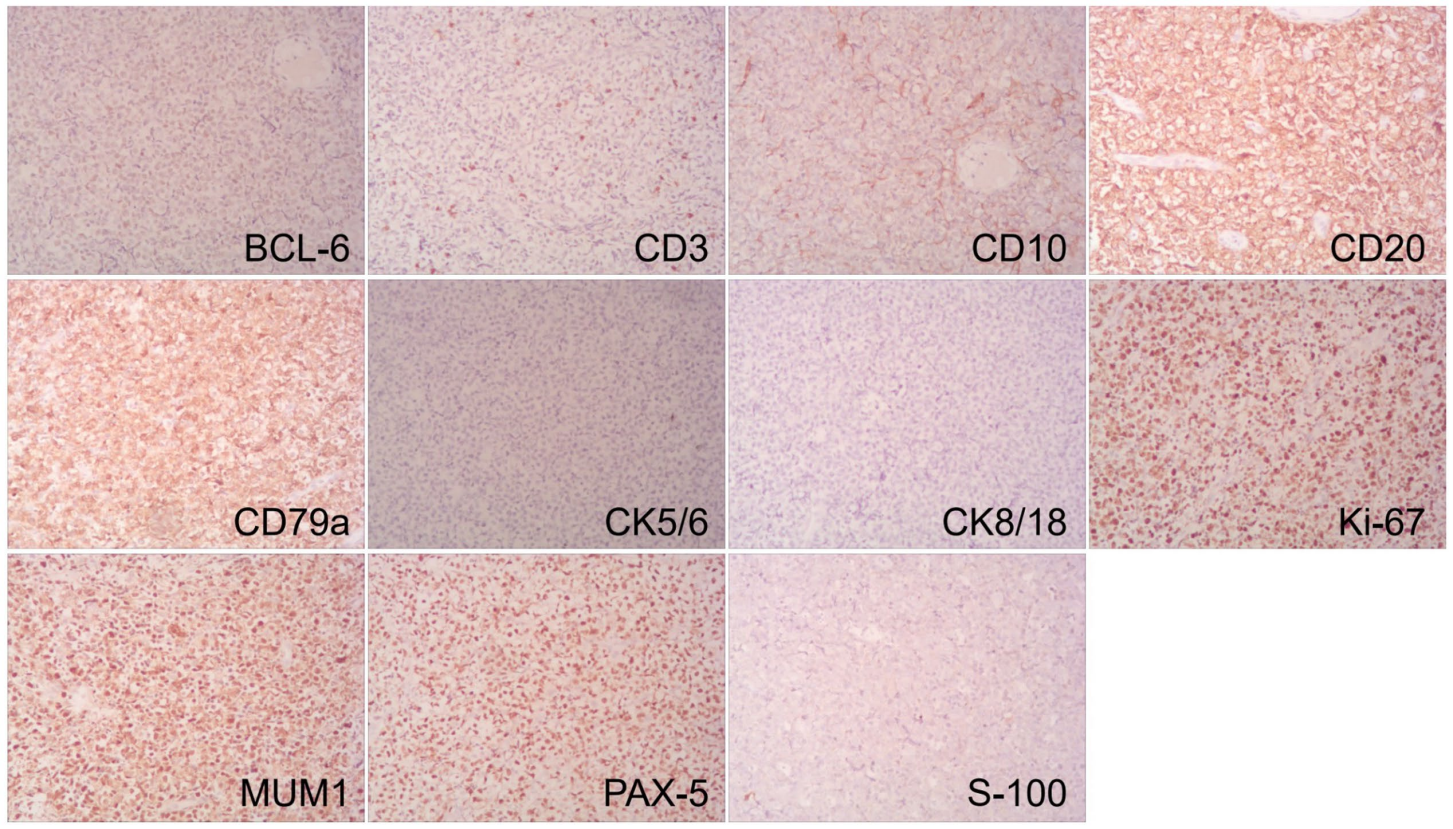
Immunohistochemical staining results of the lesion tissue. The positive and negative expression of related markers (Bcl-6, CD3, CD10, CD20, CK5/6, CD79a, CK8/18, Ki-67, PAX-5, MUM1, S-100) in tumor cells.

On February 4, 2024, the patient underwent debridement and autologous skin grafting of the right upper extremity under general anesthesia. Postoperative pathological examination confirmed the diagnosis of non-Hodgkin’s lymphoma, primary cutaneous diffuse large B-cell lymphoma, leg type, non-germinal center origin (PC-DLBCL-LT, non-GCB). Immunohistochemical staining results (Figure 2): CK5/6 (−), P63 (focal +), CD3 (T cells, +), CD20 (+), CD79α (+), BCL-2 (+), CD45RO (T cells, +), Ki-67 (80% +), CD10 (focal +), BCL-6 (−), MUM-1 (+), PAX-5 (+), CD30 (−), S-100 (punctate +), HMB45 (−), MelanA (−), CK8/18 (−).

## 3 Treatment

After admission to the Department of Orthopedics, the patient completed routine preoperative examinations including the four infection markers, four coagulation function tests and plain chest computed tomography (CT) scan. He underwent debridement and tumor lesion resection of the right upper extremity under brachial plexus block anesthesia on January 29, 2024. With the postoperation pathological findings, the Department of Orthopedics organized a multidisciplinary team (MDT) consultation involving the Departments of Hematology-Oncology, Pathology, Radiology and Dermatology. After thorough discussion, the consensus was reached to perform the second debridement followed by autologous skin grafting. The autologous skin grafting operation was done on February 4, 2024. The postoperative wound healing was good without obvious complications. The patient was subsequently transferred to the Department of Hematology-Oncology in March 2024 for planned systemic chemotherapy.

**Figure 3:**
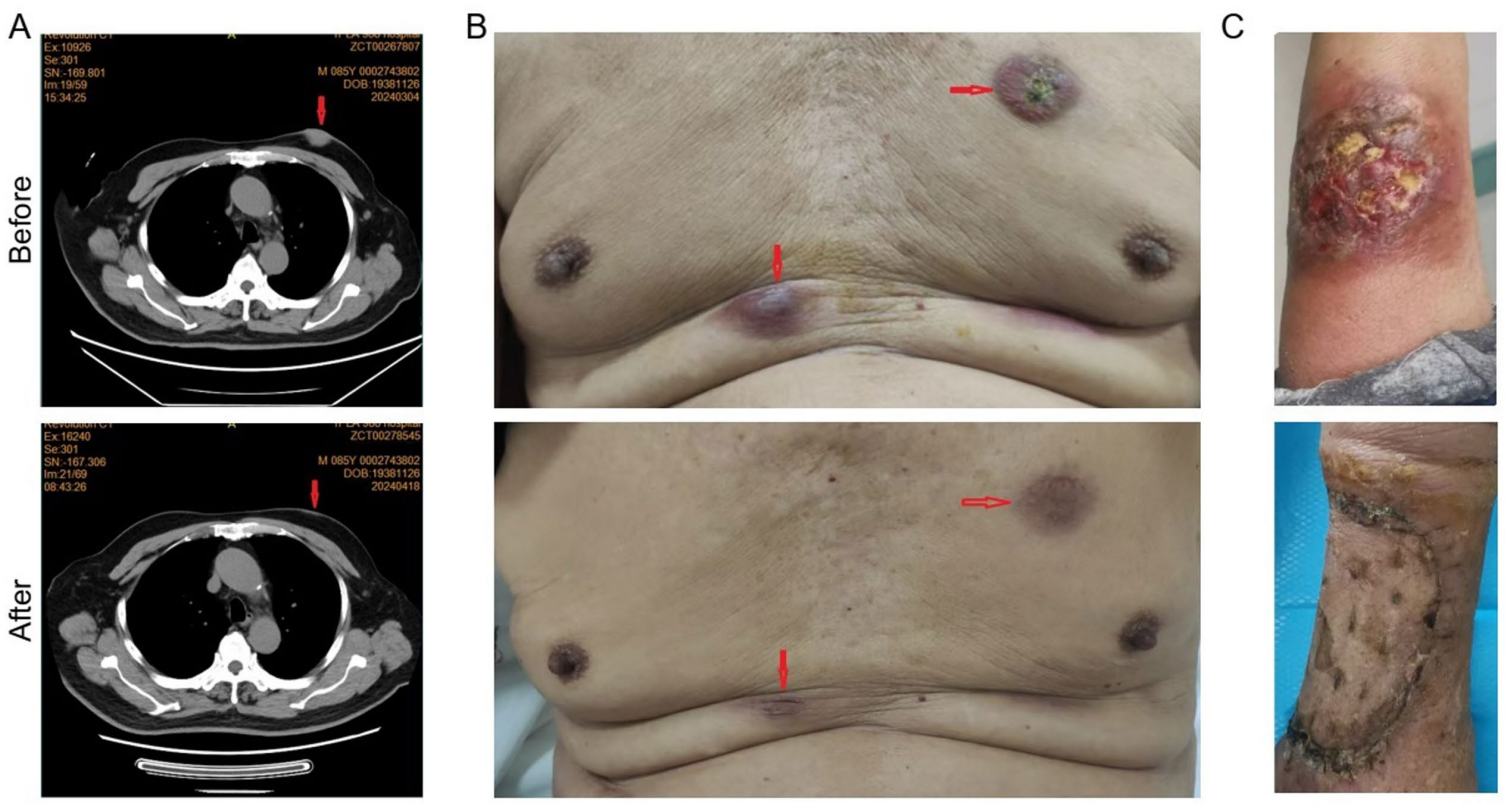
Chest CT scan results and skin recovery of the patient. **(A)** CT images of skin nodules on the anterior chest; **(B)** Photos of skin nodules on the anterior chest; **(C)** Photos of skin ulcer and flap transplantation in the right forearm.

*Reexamination results on admission to the oncology department*: chest CT scan on March 4, 2024 showed new subcutaneous soft tissue nodules on the left anterior chest wall and localized thickening of the bilateral abdominal wall skin (red arrow in Figure 3A), the largest cross-section was approximately 15.8 mm × 25.1 mm with a CT value of about 47 Hu, clear boundary and irregular shape. Bone marrow aspiration cytology showed a markedly hyperplastic bone marrow picture without obvious tumor cell infiltration.

Combined with the medical history, clinical manifestations and relevant examination results, the definitive diagnosis was made as: Primary cutaneous non-Hodgkin’s malignant lymphoma (diffuse large B-cell type, non-germinal center origin, CD20+).

The patient received 4 cycles of systemic chemotherapy with the R-miniCHOP regimen on March 7, 2024, March 28, 2024, April 19, 2024 and May 10, 2024.

*Specific drug dosage and administration method*: Rituximab (RTX) 700 mg, intravenous drip, d1; Cyclophosphamide (CTX) 600 mg, intravenous drip, d1; Vincristine (VCR) 1 mg, intravenous drip, d1; Prednisone (PDN) 40 mg, oral administration, d1-d5.

*Reexamination during chemotherapy*: chest CT scan on April 18, 2024 showed localized thickening of the left anterior chest wall skin, the subcutaneous nodules on the chest wall were significantly reduced compared with the CT scan of March 4, 2024, and the original thickened skin nodules on the bilateral chest wall completely disappeared (Figure 3B).

**Figure 4:**
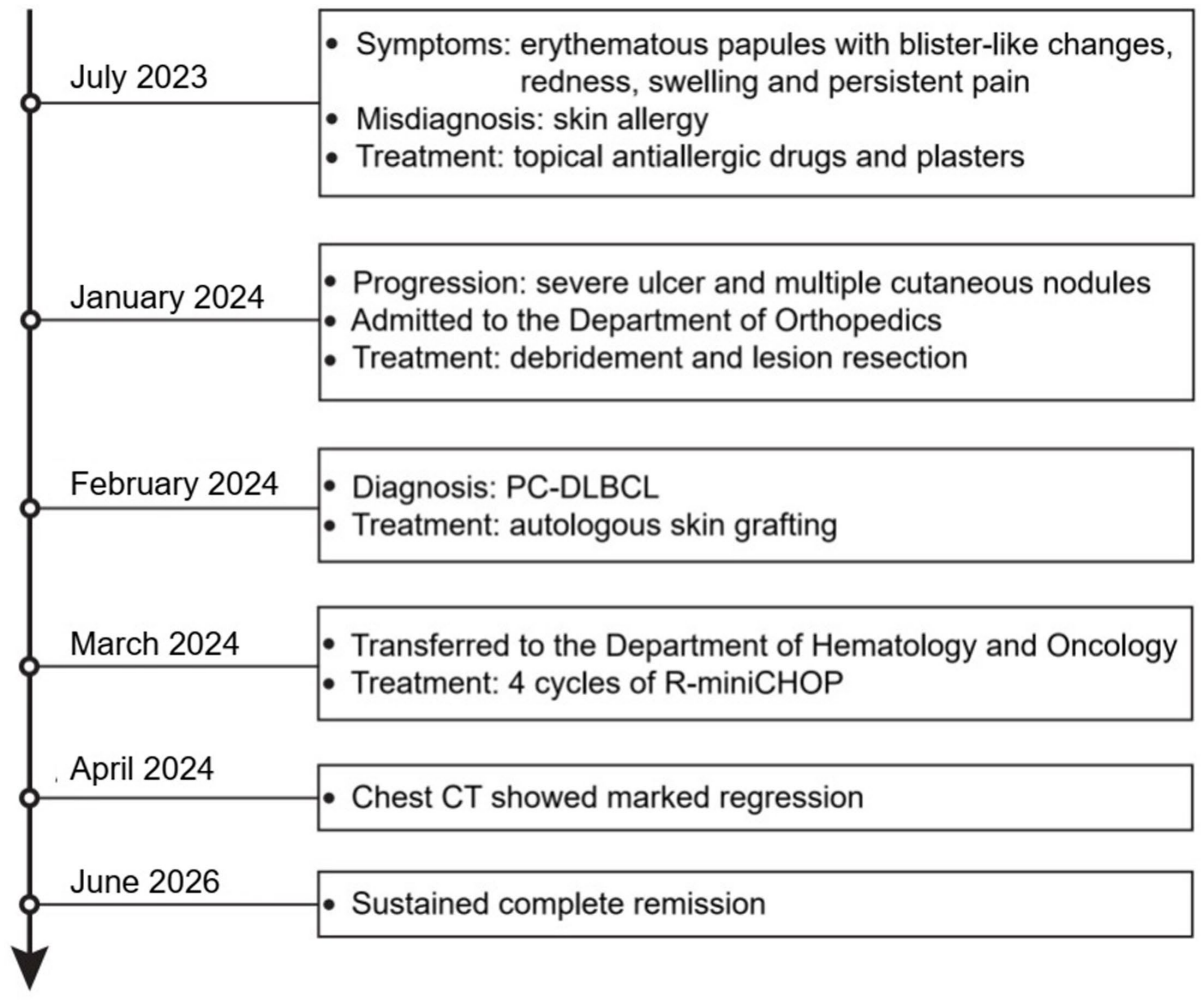
Patient clinical course timeline.

*Efficacy evaluation after 4 cycles of chemotherapy*: the skin surface nodules on the anterior chest, back and left upper arm dried up and fell off with complete epithelialization of the ulcerated surface (Figure 3B); the skin ulcer in right forearm recovered well after skin flap transplantation with good wound healing and no local recurrence (Figure 3C). The patient and his family requested discharge for follow-up treatment at home due to personal reasons. A telephone follow-up on June 1, 2026 showed no new skin lesions, no discomfort symptoms and a good general condition of the patient. The patient felt satisfied with the treatment (Figure 4).

## 4 Discussion

Non-Hodgkin’s lymphoma (NHL) is a heterogeneous group of lymphoid malignancies driven by abnormally differentiated and proliferated lymphocytes (predominantly B cells), which is more common in elderly males. These abnormal lymphocytes lose their normal anti-infection function and eventually form local or systemic masses due to unrestricted proliferation (4, 5). As a rare subtype of NHL, cutaneous lymphoma originates from the skin and/or mainly invades the skin tissue (6). According to the 2018 WHO-EORTC and the 5th WHO hematopoietic neoplasm classification, cutaneous lymphoma is subclassified into primary cutaneous lymphoma and secondary cutaneous lymphoma, which is further divided into cutaneous T-cell lymphoma and cutaneous B-cell lymphoma with multiple independent pathological subtypes, respectively.

Skin serves as the largest physical and immune barrier against the external environment, and can produce lymphoproliferative lesions when subjected to physical, chemical or biological stimuli. Distinguishing reactive lymphoid hyperplasia from malignant lymphoma remains clinically challenging owing to overlapping morphologic features. In addition, the complex pathological classification of cutaneous lymphoma further increases the difficulty of accurate diagnosis. Primary cutaneous malignant lymphoma has a low incidence in clinical practice with few relevant domestic and foreign reports (7). In this case, the patient was misdiagnosed as skin cancer and pemphigus in local hospitals and administered topical cytotoxic drugs such as nitrogen mustard, an outdated alkylating cytotoxic agent not recommended for primary cutaneous lymphoma. This treatment induced progressive full-thickness cutaneous ulcer penetrating subcutaneous tissue complicated by secondary bacterial infection, aggravating patient suffering.

NHL cells have relatively consistent morphological characteristics: medium cell size, mosaic or cobblestone arrangement, square cytoplasmic boundaries, round nuclei, clumped chromatin, multiple basophilic nucleoli near the nuclear center, and a high mitotic rate (8). Different types of lymphoma have varying degrees of overlap in cell morphology, immunophenotype and genetic aberration, which brings great challenges to the pathological diagnosis. It is necessary to select appropriate immunohistochemical staining markers, auxiliary labeling with fluorescence *in situ* hybridization (FISH), and even next-generation gene sequencing (NGS) to make a final specific subtype diagnosis (8). For this patient, the Department of Pathology of our hospital performed a comprehensive immunohistochemical staining with a series of markers on the basis of conventional HE staining, which further clarified the pathological type and immunophenotype of the skin lesion and pointed out the direction for the subsequent targeted treatment. Therefore, the diagnosis of lymphoma is highly dependent on pathological examination. Accurate pathological typing lays a solid foundation for standardized treatment. The poor therapeutic response of the patient in local hospitals was mainly attributed to sole empiric therapy without pathological examination.

The key clinical point to distinguish primary and secondary cutaneous lymphoma is whether there are lesions in other systems except the skin at the time of initial diagnosis (9). Primary cutaneous lymphoma presents isolated skin involvement without extracutaneous organ/lymph node infiltration at baseline workup, whereas secondary cutaneous lymphoma presents cutaneous dissemination from pre-existing systemic lymphoma. This patient was finally diagnosed PC-DLBCL-LT, which is a relatively rare subtype with no obvious systemic lesions at the time of diagnosis.

Macagno et al. (10) pointed out that the treatment plan of cutaneous lymphoma is closely related to the clinical stage: supportive therapy and local treatment (such as local radiotherapy, topical drugs) are the main treatment measures for early-stage diseases, while systemic combined chemotherapy combined with immunomodulator therapy is the first choice for advanced-stage diseases with systemic involvement. According to NCCN guideline v2026, treatment of PC-DLBCL-LT is stratified by disease dissemination. Solitary regional (T1–2): Pola-R-CHP + Local ISRT or Local ISRT or clinical trial is recommended. Local ISRT; Generalized disease (skin only): Pola-R-CHP + Local ISRT or clinical trial is recommended; Extracutaneous disease was recommended to be managed as DLBCL (11). Consistent with above international criteria, this elderly complicated patient was treated with rituximab-based molecular immunotherapy combined with chemotherapy with reference to the treatment principles of B-cell NHL. Rituximab injection is a chimeric monoclonal antibody targeting the transmembrane antigen CD20, which can specifically bind to the CD20 antigen on the surface of B lymphocytes, initiate and mediate specific lysis of B lymphocytes, and inhibit the abnormal proliferation of B lymphocytes (12).

In this case, after 4 cycles of rituximab combined with the mini-CHOP chemotherapy regimen, the proliferative lymphoma nodules on the patient’s chest wall and abdominal wall shrank significantly, the ulcerated cutaneous lymphoma nodules gradually dried up and fell off with complete epithelialization of the ulcerated surface, and the right arm skin recovered well after skin flap transplantation. The telephone follow-up 12 months after systemic treatment showed the patient was in good condition with no recurrence, indicating a satisfactory long-term therapeutic effect.

In conclusion, PC-DLBCL is a rare subtype of NHL with rapid progression and non-specific clinical manifestations, which is prone to be misdiagnosed and mistreated in clinical practice. For elderly patients with unexplained skin nodules, ulcers and poor therapeutic effect after conventional treatment, pathological examination should be performed as early as possible to clarify the diagnosis. Accurate pathological diagnosis and timely standardized comprehensive treatment are key to improving the therapeutic effect and prognosis of patients.

## Data Availability

The original contributions presented in the study are included in the article/supplementary material, further inquiries can be directed to the corresponding author.
